# Texas Cattle Fever*Read before the American Public Health Association at its annual meeting at Detroit, November 14, 1883.

**Published:** 1884-07

**Authors:** D. E. Salmon

**Affiliations:** Veterinarian U. S. Department of Agriculture


### THE JOURNAL

OF

### COMPARATIVE MEDICINE AND SURGERY.

VOL. V. JULY, 1884. No. 3.

### ORIGINAL COMMUNICATIONS.

### ART. XV.—TEXAS CATTLE FEVER—IS IT A CHIMERA OR A REALITY?*

BY D. E. SALMON, D.V.M.,

*Veterinarian U. S. Department of Agriculture.*

As long ago as 1796 there was an outbreak of cattle disease in Lancaster county, Pa., which was attributed to infection from a drove of cattle previously brought from the State of South Carolina. In every instance where these animals had mixed with others a disease was contracted, and in one case this disease was supposed to have been induced indirectly by the grounds on which the Southern animals had been penned.

It is extremely interesting from our standpoint to note the peculiar characters of the disease as observed at that time; for then there was no previous tradition to lead them to ascribe such an affection to Southern cattle. The outbreak occurred in the month of August; there was weakness of the

* Read before the American Public Health Association at its annual meeting at Detroit, November 14, 1883.

limbs, amounting to inability to stand; when the animals fell they would tremble and groan violently; some discharged bloody urine; the bowels were generally costive; the kidneys, on *post-mortem* examination, were found inflamed.

Since that time there have been many outbreaks of disease in Pennsylvania, in Maryland, in Virginia, in North Carolina, in Georgia, in Tennessee and in Alabama, which I cannot particularize at this time; but which were attributed to infection brought by apparently healthy cattle that had come from towards the Atlantic or gulf coasts. These outbreaks invariably occurred in summer; they were characterized by weakness of the limbs, constipation, bloody urine, drooping head and lopped ears. The duration of the disease was from three days to a week; the sick cattle seldom, if ever, infected other animals; on *post-mortem* examination the most conspicously diseased organs were the spleen and kidneys. The plague stopped its ravages with the first heavy frost.

It was not until 1853 that similar accounts began to come from the South-west and West, and as in succeeding years a greater number of cattle were driven from Texas into Missouri, Kansas and Iowa, so the cases of infection charged against them multiplied enormously. So certain were the people of these States, after a few years' experience with the disease, that it was brought by the Texas cattle, that in 1861 laws were enacted to regulate the movements of the Southern herds.

This seems to have been done in complete ignorance of the fact that the same disease exists in the Atlantic States of the South, and although similar laws had been framed in North Carolina as long ago as 1837, the laws of the West were the result of an independent experience and local necessity.

At this time, however, the war broke out, the driving of cattle from the South ceased, and the disease disappeared only to return with the first Texas droves.

It is not my purpose to recount to you the many fatal outbreaks which occurred during the years 1866, 1867, and 1868, when Texas cattle were carried without restraint into the very heart of the great stock-raising sections of the West. They are on record elsewhere, and some of the honored members of this Association investigated them and deserve great credit for what they did towards giving us correct ideas of this scourge. Within two miles of the Chicago stock-yards 161 animals perished in a few days during the summer of 1868; in a single township 926 head of cattle were swept away; on a single farm more than 400 others contracted the disease and died.

These examples are all taken from the State of Illinois; but Ohio, Indiana, Kentucky, Iowa, Missouri and Kansas suffered enormously as well, and in all cases the animals which died had been upon pastures or grounds which had previously been occupied by Texas or other Southern cattle.

This great outbreak of 1868 led to a number of investigations, among which we may refer particularly to the valuable work done by the Metropolitan Board of Health, by the U. S. Department of Agriculture, and by the Chicago Board of Health. In every case a disease was described having substantially the same character—drooping head and ears, weakness of limbs, constipation, bloody urine, high temperature, ending in death after three or four days, or even longer. And in every case the disease was supposed to have been caused by Texas cattle. The alarm produced by the terrible losses of 1868 was such that the movements of these animals have since been regulated, and comparatively few have been carried directly from Texas to the pastures of the North, though there is never a year but that some such cases occur, and we learn of them by the disease which invariably attacks the native cattle.

It is not the Texas cattle alone which are charged with spreading this fatal disease, though the enormous losses caused by these animals from 1866 to 1868 were sufficient to link the name of Texas indissolubly with the malady. I have already referred to outbreaks caused by South Carolina cattle, and if we make a thorough inspection of the Southern States we will find a vast district, stretching from the Rappahannock in Virginia to the Rio Grande, which has the reputation of sending forth cattle that, though remaining healthy themselves, are endowed with a mysterious power of spreading disease and death wherever they are carried. I could detail to you instances of this kind which have occurred in Virginia, North and South Carolina, Georgia, Alabama and Tennessee, sufficient to fill a volume—cases observed independently of each other, but in which the same conclusions were reached with a unanimity that is really surprising.

Having studied the apparently reliable investigations of others to whom I have referred, having gone over much of the Southern territory alleged to be infected with this disease, and having traced many outbreaks to cattle from this district, you may judge of my astonishment when I read the conclusions which were reached by a member of this Association who seems to have devoted considerable time to a study of the subject.

Dr. Smith has "been trying," he tells us, "to ascertain whether the cattle of Texas are habitually unhealthy or diseased; whether any such disease prevailed among them as made them dangerous as sources of infection if driven to Northern markets; and to find out whether it ever occurred that these cattle, being themselves healthy, could nevertheless communicate not only once, but habitually, disease to healthy cattle with which they came in contact." And from these investigations he concludes that "it certainly seems an error to suppose that any danger is incurred by the transportation to Northern markets of Texas cattle."

In other words Dr. Smith would have us believe not only that all the losses attributed to Texas fever have been caused by some other disease, but that the Texas or Southern cattle have not brought the infection and are not responsible for the damage. On the other hand, my own conclusions are diametrically opposed to such a view; but this alone would hardly have lead me to contest the matter as I am now doing. It is only when I consider that a very large territory is now overrun with the permanent infection of this disease, that this infection is continually advancing, and that hundreds of thousands of dollars are annually lost through ignorance of its character, and that it can only be held in check by rigorous sanitary laws, that I determined to present my views to this influential body in such a form as to leave no occasion for a misunderstanding.

Dr. Smith furnishes three lines of observations by which he attempts to establish the innocuousness of Texan cattle, and from these he concludes:

1st. That Texan cattle killed for food are generally free from pathological lesions.

2d. That the native Texan cattle "are singularly free from disease."

3d. "That the sickness and mortality among the imported cattle are due to an acclimating process."

These conclusions, it seems to me, are not only based upon insufficient evidence, but they could never have been reached without excluding and ignoring a very important part of the facts, which you will find in the papers which he has presented to this Association. To show that such is the case, I will review these papers with sufficient detail to justify this assertion.

First, as to the *post-mortem* appearances. Dr. Smith's report of 1881 contains particulars in regard to 250 animals which were examined at fourteen different places by fifteen observers. Two of these found bloody serum in the pericardium in a certain number of cases, twelve as stated in the table, though there seems to be some doubt about the number.

Dr. Buffington found fatty degeneration of the liver in four out of fifty animals. The microscope was not used, but he was satisfied of its existence from the color and general appearance. In eight cases the gall bladder was reddened.

Dr. Gorgas reported: "The livers in all cases were markedly yellow and somewhat softer than they should be. The color had the appearance of bile staining."

When we remember that one of the most frequent lesions of Texas fever, as seen at the North, is a softening and yellow coloration of the liver, due, as Dr. Stiles demonstrated, to repletion of the biliary radicles and to yellow staining, the result of a mixture of blood with the bile, we are in a position to appreciate the significance of the yellow coloration noticed by those two observers.

The appearance of the spleen is of even more interest to us, because it is the organ which suffers beyond all others in Texas fever. It seems to be admitted that the spleen averages a greater weight in Texan than in Northern cattle, but I do not care to lay great stress on this indication; it can at best have but a negative significance. Not so, however, with individual spleens so heavy as to suggest disease.

In one cow this organ weighed five and three-quarter pounds, and was associated with bloody serum in the pericardium, with a "spotted" condition of the mucous membrane of the stomach and a reddened bladder. Two other spleens weighed over five pounds, and four between three and four pounds. In a case where the spleen weighed five and one-eighth pounds, it was described as "pulpy," the pelvis of the kidneys was "streaked," the liver had the appearance of fatty degeneration, the gall bladder was reddened, the lining membrane of the bladder showed hemorrhage, and that of the fourth stomach petechiæ.

The consistency of the spleen was described differently by the different observers. One says, inclined to flabbiness; another says, soft, brittle, easily broken down and very vascular; a third says normal; a fourth calls it the consistence of jelly; a fifth, pulpy; a sixth, doughy rather than firm; a seventh, rather soft and brittle, and four others firm, one adding solid. Dr. Smith adds: "It would seem, in reference to consistency and color, that different observers, according to their particular idiosyncrasy, described the same condition by different terms." For my part I cannot look at the matter in this way. It is impossible to conceive that a spleen which would appear to one medical man to be firm and solid, would appear to another competent observer to be soft, brittle, easily broken down, pulpy, or having the consistency of jelly. The appearance described by the latter class of terms is so similar to what is seen in acute cases of Texas or Southern fever that it seems more than probable that four or five of the above observers described organs more or less affected by the disease.

We are the more inclined to this view because of the lesions associated with these abnormal appearances. In one case the spleen was spoken of as "dark colored," it weighed five and three-quarter pounds, and there was at the same time bloody serum in the pericardium; the lining membrane of the fourth stomach was "spotted," and the bladder was "light red." In a second case, the spleen weighed five and one-eighth pounds, and the membrane of the fourth stomach was "spotted." In a third case the spleen was "pulpy," the liver yellow, the pelvis of the kidneys "streaked," the lining membrane of the bladder showed hemorrhage, and that of the fourth stomach petechiæ. In a fourth case where the spleen was dark purple and weighed two and three-quarter pounds, the mucous membrane of the bladder was reddened, and that of the fourth stomach showed petechiæ. Nine out of twenty examined by one observer had petechiæ of the mucous membrane of the fourth stomach; while in sixteen out of sixty-four seen by another observer, the same part was "spotted."*

In the face of these facts it is impossible to admit that the different observers, according to their particular idiosyncrasy described the same condition by different terms, and we must conclude, as well from Dr. Smith's facts as from those presented by others, that a part of the cattle killed for food in Texas shows lesions similar to, though less intense, than those seen in the fatal cases of Texas fever.

I don't care to dwell upon this fact, as the present condition of our knowledge is not such that we can thoroughly define its meaning. We have no means of knowing whether it is necessary for Southern cattle to show lesions of disease in order to communicate it, or whether it is possible for them to distribute infection when we are unable to find abnormal appearances either before or after death. It is sufficient for my purpose to establish the fact that a very considerable proportion of Texas cattle, apparently in the best of health when killed for food, present appearances similar in character to those seen in fatal cases of the so-called Texas fever at the North.

The next point to which I beg leave to direct your attention is as to whether native Texan cattle suffer from any disease similar to that known at the North as Texas fever. Texas people at present are very sensitive on this point, and where the question is answered in a general way it is not always answered with absolute exactness.

Dr. Smith has not furnished us with many reports in regard to loss among Texas cattle, and these few vary greatly and are extremely indefinite, yet they scarcely support his conclusion that but a small portion of Texans die, and these mostly old cows, from being unable to stand the winters. We are told that at Presidio del Norte, while there is "no prevailing disease of any kind," it has happened that two or three times during the last fifteen years a large proportion of the herds about the Cheuati Mountains and upper Chihuahua, died almost suddenly without apparent cause, from a disease that affected the liver. The principal cattle raiser in the town of Del Norte, "a tolerably intelligent Mexican," places first in his list of prevalent diseases one of an epizootic nature, which prevails in the fall, and of which the characteristic symptoms are wasting, loss of appetite, and bloody urine. If this is not Texas fever will some one mention another disease to which the description will apply? The second disease is also epizootic—the symptoms are quietude, tendency to droop the head, loss of appetite and dryness of the horns. This I suspect is but a different type of the same disease. The third disease mentioned is undoubtedly black quarter—the *charbon symptomatique* of the French.

"Mr. Rooney has one thousand head of cattle on the Pecos river. He lost fourteen head of cattle last year and fifty-four head this year. The disease has been among his cattle for three or four years. He made a *post-mortem* examination of one animal and found the gall bladder filled with material black and thick as tar. Bladder distended with blood. The disease lasts about three days. Symptoms: the animal is sleepy; froths at the mouth." This description may not be entirely satisfactory, but in the distended gall bladder, the bloody urine and the duration of the disease, I recognize the lineaments of our old acquaintance.

Mr. Richards on the Pecos lost fifty head last year and fifty head this year (I take all these cases from Dr. Smith's papers,) from a disease which prevails most in October, and in which "the spleen was black and soft."

Mr. Kieeling, also on the Pecos, lost eighty-seven in one season and found the bladder filled with "black fluid."

Mr. Johnson lost two head; "the animals urinated blood and the bladder was filled with blood."

In some of these cases the disease was attributed without evidence to poisonous weeds.

Dr. Gerard heard of a certain "murrain" which killed a number of cattle near Fort Stockton two or three years ago. The term murrain I have learned from experience is used from

Georgia westward to designate the disease from which cattle die when imported from the North—in other words, it is synonymous with Texas fever, and hence this report conveys an impression to my mind which it does not to the casual reader.

These cases then look very like Texas fever, and they are about as numerous as we should expect to have developed by an inquiry of this kind in a section where the native cattle unquestionably possess an extraordinary degree of insusceptibility to this disease, and where the people believe it to their interests to suppress the facts.

I will add a little information to this, however, which I trust may make the matter as clear as can be desired. In Special Report No. 22, U. S. Department of Agriculture, I published a letter originally written to the *National Live Stock Journal*, in which the following sentence occurs: "In the section of the country known as the Pan-Handle of Texas I might make a fair estimate by saying that one thousand head of cattle die annually from that disease," *i. e.*, Texas fever.

In Special Reports Nos. 12, 22 and 34 we have published information in regard to diseases by counties, and I would mention particularly the following, which refer very plainly to the disease under consideration:

"Camp County—Several cattle have died of bloody murrain.

"Dallas County—Cattle die mainly of bloody murrain.

"Hopkins County—Cattle are affected with bloody murrain.

"Navarro County—Cattle are frequently attacked by bloody murrain, but I do not regard this as an infectious or contagious disease.

"Titus County—Cattle are affected with murrain and black tongue.

"Uvalde County—Cattle are mainly affected by some kind of slow fever known generally as Spanish fever, (a synonym of Texas fever.)

"Walker County—Murrain is very prevalent among cattle, and but few attacked recover."

During the present year a gentleman at Dallas, Texas, wrote as follows to the *Rural New Yorker:* "For several years we have lost cattle at intervals in spring, summer and fall by what is here called bloody murrain by some, and Spanish fever by others. So far every case has proven fatal. Symptoms—First, nose and horns cold, with dysentery; after a few hours the affected animals pass from the bowels a dark mucous steaked with blood. The urine is a bright red, looking as if half blood; sometimes the beasts appear in great pain, while at other times they are quite."

In the month of August of the present year the Department of Agriculture received a letter from Dr. Gardner, Major and Surgeon of the U. S. Army, of Fort Davis, Texas, asking for information in regard to a very serious contagious or epizootic disease among the cattle in that vicinity. Dr. Gardner did not apparently suspect that the disease was Texas fever, and this affection was not alluded to in his correspondence; but he named another and a very different malady as the one which he supposed was responsible for the losses. I mention this because it is important to understand that this description, though familiar enough to some of us, was entirely original with him. The disease appeared about the fifth of August; the animals were sick about three days before they recovered or died; probably one-half the cases were fatal, and the twentieth of August it was stated that many hundreds of cattle had already died. Later we received a letter with fuller particulars from which I extract the following:

"About the first of July a herd of sixteen hundred cattle from somewhere along the gulf coast debarked from the Southern Pacific railroad at Murphysville station, making northward through this section. * * * I have heard it stated that before this herd of cattle left their gulf coast range the most of them were sick, and many had died. It is uncertain whether any of this herd died while *en route* through this section. It is, however, certain that in a few weeks cattle kept upon ranges through which this herd passed were found to be sick; the epidemic spread all through the herds kept on ranges crossed by their line of march, and in many cases the disease was very fatal. One freighter on the road (in Lymphia Canon) losing seven individuals out of a total of nine attacked. The disease appeared to me to be highly contagious, but not epidemic.

"I had opportunities of observing the disease in several living animals, and noted the following symptoms: The sick animals were weak and staggered in their gait; the pulse was frequent and feeble; the inspiration was shallow, hurried and panting; * * * the alvine evacuations were either scanty and dry or else profuse and watery; the urinary secretion was either entirely absent or profuse and bloody; * * * I am under the belief from the action of the animals that there was more or less delirium in every case. * * *

"I was only able to make *post-mortem* examinations of the bodies of four individuals that died of the disease. In every instance the spleen was found to be enlarged, elongated, dark purple, almost black and friable; the liver was enlarged and congested, and the gall-bladder filled with a thick dark green, semi-crystalline bile; the kidneys were enlarged and congested, and in every instance the urinary bladder was filled with dark bloody urine."

This outbreak had already abated by the 28th of August.

Here is a wonderfully accurate portrayal, from one who supposed he was describing an entirely different disease, of what is usually seen in an outbreak of Texas cattle fever. There is the drove from the Gulf coast, driven to the mountainous district of Southern Texas, probably entirely beyond the section permanently infected with this disease; it crosses the ranges of healthy cattle, which in a course of a few weeks begin to sicken by the hundred; the course of the disease is rapid, yet not sufficiently rapid for anthrax or charbon; the malady sweeps through the herds like a fire over the Western prairies, and in a few weeks all is over. Yet it does not spread from herd to herd, it is only those animals which have crossed the deadly trail of the Gulf coast cattle that are affected.

In this connection I wish to direct your attention particularly to a fact that seems to have escaped the notice of others. Texas is an extremely large State: it embraces within its territory the most varied characters of soil and climate. In the Southeast there is the low, flat, unhealthy Gulf district one hundred miles or more wide: then comes an undulating, hilly belt of an entirely different aspect and character two to three hundred miles across; finally, in the West and Southwest there is a mountainous district of an almost equal width. It is only within recent years that the stockmen have crossed the middle belt and have found their way to the Western. We have long had the best of reasons for believing that the Eastern and Southeastern parts of Texas were permanently infected with this disease, but until very recently we knew nothing about the Western half of the State, because comparatively few cattle had been raised and driven from there. But if, as our most recent reports indicate, cattle can be taken from the Northern States to the Western part of Texas and remain there without contracting this disease; if droves from Eastern Texas carry the infection there and destroy the native cattle the same as they do at the North, then it follows that the cattle of Western Texas do not possess an immunity from this disease, that the ranges of that section are not yet permanently infected with it, that we have done injustice to Texas in attributing an evil to the whole State which only exists in a part of it. And this may help to explain to us why the spleen of animals killed for food at San Antonio and Fort Ringgold in the East were of the "consistence of jelly" or "all pulpy," while in the West they were firm and solid; it may also explain why no epizootic disease was reported from the East where the cattle are insusceptible, and why we get accounts of such at Presidio, at Fort Davis, at Fort Stockton, and at various points on the Pecos river. It also suggests the inquiry if it may not be as important to Texas as to any State in this Union to recognize the existence of this disease, to mark out the permanently infected district, and to take timely measures to stop the annual infection of her Western ranges and herds, and what is of more importance still, to check the encroachment of the permanently infected district upon them.

It is unnecessary for me to bring more evidence bearing on this point. I simply desire to prove to you that Texas cattle on their native ranges are affected with a disease having all the essential characters ascribed to the disease that they have been charged with disseminating among Northern herds. From an investigation extending over the past four years I know that the native cattle occasionally die from it in all parts of the permanently infected district of the South; but I know equally well that such cattle are very insusceptible to it, and that it seldom assumes epizootic characters among them. If they did not possess this immunity nine-tenths of the adult bovine population of the South would perish in a single summer. When, therefore, I defined this disease as "an exceedingly fatal epizootic," a definition to which Dr. Smith seems to have some objections, I referred, of course, to its effects upon susceptible cattle and not upon those which in some way have acquired the power of resisting it. I might truthfully say that yellow fever is a terribly fatal affection, although as is well-known the inhabitants of Cuba who have recovered from one attack expose themselves to the disease with comparative safety; and yet the mortality from the most virulent attacks of yellow fever scarcely exceeds half of what we see with Texas cattle fever.

I have no desire to exaggerate the losses among the native cattle of the South; I will even admit that they are surprisingly small considering that the whole district is saturated with the germs of so deadly a plague—my object at present is simply to combat the idea that Texas cattle can be safely scattered over the great live stock sections of the North. And with this object in view, I care not if Dr. Smith were able to prove, which he is not, that a case of Texas fever has never occurred among the native cattle of Texas; and I care no more if he were able to prove, which he is not, that among all the cattle killed for food in that great State, not a single one presents the lesions of this disease; I am one of those who believe that we, as sensible men, must accept a fact, when it is demonstrated to be a fact, whether we as scientific men have a satisfactory explanation for it or not. It has been quite the fashion for medical men to say that it does not look reasonable that a healthy, or an apparently healthy steer could disseminate so fatal an infection; and again it does not look reasonable that animals which have been infected in this way should not in their turn infect others. I contend, however, that as scientific men we are oblige to face the facts, that we have no business to speculate as to the reasonableness of a phenomenon, but that it is our duty first of all to ask: Does it occur, or does it not?

The third conclusion in Dr. Smith's paper which I am forced to contest, is that the great mortality which attends the importation of cattle from the North, is due to an acclimating process in any proper sense of the term. So far from this being the case, I am satisfied from my observations that the great majority of such animals die from the disease called Texas fever, and that the change of climate has little or no influence on the result.

Consider, if you please, that cattle are frequently taken from Canada to Kentucky, Missouri or Kansas without any such disease resulting, while it is uniformly produced in those animals transferred from Kansas to Texas, from Missouri to Arkansas, from Tennessee to Mississippi, Alabama or Georgia; and yet, the changes in the latter cases are not nearly so extreme as in the former. But it is not necessary to move cattle as far as this, even, to have just as fatal results as with those carried from New York to Texas. Beef-cattle which go from Northern Georgia to Savannah are affected to such an extent that they are only shipped in winter, and even then so many are affected before they are killed, that I have been informed the Board of Health has been considering if it would not be advisable to shut out such animals entirely, though they are the only really good beef which goes to that market.

Animals shipped from Northern Georgia to Charleston suffer to an equal degree. So animals going from Middle Virginia to tide-water Virginia in the same latitude; from west of the Blue Ridge mountains in North Carolina to the middle section of the same State; from less than a hundred miles north of Atlanta to that city, are afflicted to the same extent. But what bears even stronger against the acclimation theory is the fact that animals which simply cross from the North to the South bank of the James river in a part of its course, or from the North to the South bank of the Staunton river in a part of its course, or from the North to the South bank of the Yadkin river, in a part of its course, suffer with the same symptoms and die in the same proportion as those which have been transported a thousand miles. It is not, then, the great difference in summer heat, in malarial emanations, in the character of the herbage and soil, that induces the mortality among cattle taken to the

South, since it occurs where the animals have been moved too short a distance to experience such changes.

The second consideration bearing against this conclusion is that the district in the South to which it is dangerous to take imported cattle is precisely that from which the native cattle carry infection when driven North. I have not yet investigated the infected district west of the Mississippi, but I have spent much time in locating it in the States east of the river, and I am satisfied that I can present this statement as a general law. I have traced losses in the counties directly north of the James river to cattle which came from directly south of it; I can say the same of the Staunton river; of the Blue Ridge mountains rising East and West instead of North and South; the same in regard to cattle carried from county to county in parts of Virginia, North Carolina, Georgia, Alabama and Tennessee; and wherever the cattle from one county have caused losses among the native cattle of an adjoining county, there was evidence to show that cattle from the latter county when carried to the former were very certain to contract this disease. In other words, there is an infected district in the South which may be accurately outlined, and if you take cattle from that district beyond its boundary line you spread the infection, though you only move them from farm to farm; and, conversely, if you take cattle from outside of that district across the boundary line, though you only move them from farm to farm, you render them liable to the same infection. The fact that the sick animals in the latter case have not been in contact with Southern cattle proves nothing—the disease is not contracted directly from other animals, but always from infected grounds.

The third consideration bearing upon this question, and the most important of all is, that the cattle which die in the South from the so-called acclimation fever have exactly the same symptoms and the same *post-mortem* appearances as the cattle which die in the North from exposure to grounds infected by Texas cattle. Now, gentlemen, I appeal to you, is it not a sensible plan when you desire to know what is the matter with a man, or an animal either, to study the symptoms and compare them with those of familiar diseases, and, when you have an opportunity, to study the *post-mortem* appearances as well?

If you have a patient with the typical symptoms and lesions of diphtheria, I am satisfied you would pronounce it diphtheria, even though the individual had recently emigrated from Manitoba to Mexico. And so, when I see animals recently imported into the South, and I have seen many such, standing with head down, ears lopped over, back arched, walking with a weak and staggering gait and voiding bloody urine, when in three or four days they die, and after death I find erosions on the internal coat of the stomach, distended gall-bladder, enlarged and yellow liver, greatly enlarged and pulpy spleen, engorged kidneys and yellow coloration of the fat and tissues generally, I am very much inclined—indeed I am very certain to pronounce the disease Texas, Spanish or Southern fever. I consider it, then, a point too firmly established to be successfully contested that Northern cattle taken to Texas, and to many other parts of the South as well, suffer from Texas fever in about the same proportion and with the same symptoms as they would if on their native pastures they were exposed to Texan stock.

To recapitulate, I believe I have shown: First, that cattle in Texas killed for food quite frequently present lesions similar to those seen in Texas fever; Second, that Texas cattle on their native ranges occasionally suffer from Texas fever; and Third, that the great mortality acknowledged to occur among cattle imported into the South is the result of Texas fever.

After following Dr. Smith in his attempt to show that Texas cattle are remarkably healthy, that no disease prevails among them that would make them dangerous as sources of infection if driven to Northern markets, and that Northern animals imported into Texas die from the effects of change of climate, in other words that there is no such thing as Texas fever, one is somewhat surprised to find in the final paragraphs quotations from Dr. Rauch's report to prove that this disease may be transmitted from native to native cattle. In conclusion we find the following quotation, which I doubt not has had more importance attached to it than Dr. Rauch supposed it merited when he unfortunately penned it. "The assertions that native cattle die of this disease, and do not communicate it to other native cattle; that Texas cattle are perfectly healthy, and still cause disease that is fatal to native cattle, and that they do not die of this disease, are such anomalies in the history of contagious diseases that, on general principles, we could not believe them."

To this statement Dr. Smith adds: "The legitimate deductions from the reliable facts and statements contained in this present report are entirely in harmony with the views enunciated by Dr. Rauch."

I must confess there is something about this concluding paragraph of the report that I do not understand. The whole tenor of what precedes is to show that there is no such thing as Texas fever; Dr. Smith sums this up very well when he says: "If so many assertions had not previously been made and so many witnesses heretofore cited, where Texas cattle, apparently healthy, had infected other cattle, mingling with them, crossing their line of march, or following them in their grazing grounds, the inference from the foregoing would be undoubted, that there was no danger to be apprehended to other cattle by exposure to cattle from Texas."

How the "legitimate deductions" which go to show that there is no such thing as Texas fever can be "entirely in harmony" with Dr. Rauch's conclusions that Texas fever is communicable between native Northern cattle, that Texas cattle sometimes die of it and must be affected with it before they can convey it to Northern animals, is an enigma which I shall not attempt to solve. But I must protest against the assertion that the characters usually attributed to Texas fever "are such anomalies in the history of contagious diseases that on general principles we could not believe them."

Let us briefly review the so-called anomalies. First, Texas cattle possess a very complete immunity from the disease. Is that an anomaly? I take up a work on human pathology and turning to yellow fever, almost the first sentence that strikes my eye reads something like this: "Yellow fever is emphatically a disease of the unacclimated; the natives in yellow fever districts possess an undoubted immunity from the disease," etc., etc. Surely Dr. Rauch is too well informed to have been ignorant of a fact so well known and so indisputable, and, consequently, he could not have referred to this character. Secondly, Texas cattle apparently in good health carry an infectious principle to distant pastures. Is that an anomaly? I turn again to human pathology, and I read that people in apparently good health who have come from the cholera districts of the East and who have no other sign of disease than a slight diarrhœa, and who soon recover from this, undoubtedly convey cholera into uninfected countries. I read in one of the reports of this Association, "That yellow fever is frequently transported from one seaport to another by ships on which no cases of disease have occurred, and by people who are not themselves sick," (vol. 6, p. 351.) Is it very remarkable then that an animal covered with a heavy coating of hair, and having a vast alimentary reservoir containing something like a barrel of miscellaneous material gathered from infected pastures, and which is not entirely replaced for weeks, but is scattered over the grounds wherever its possessor travels—is it very remarkable, I repeat, reasoning from the facts I have cited in regard to yellow fever and cholera, that the germs of Texas fever are carried either in the hair or in the alimentary reservoirs of Texas cattle?

Once more, I am unable to see such an anomaly as to make me reject this conclusion on general principles. Sick native cattle do not, as a rule, infect other native cattle with which they come in contact. Here, certainly, we must look for the extraordinary anomaly which is to cause us to reject the great mass of testimony, not only of unprofessional, but of those professional men who have carefully studied Texas fever. Again I consult human pathology, and I find it stated by the very best authorities that "persons going from a district where yellow fever prevails into a district where it does not exist, and becoming attacked in the latter, do not communicate the disease."

Now, I would be glad to know why, if this is such an anomaly with one disease, it is not equally so with the other. When I add to this the well-known fact that Texas cattle do not convey the disease directly, but only by first infecting the pastures and runs, I think it will be apparent that even this character is not the anomaly which we were led to expect. An animal which has fed upon the infected pastures of the South, can infect other pastures, but no other animal can do this. That is apparently a simple expression of the fact—is there anything about it to cause its rejection on "general principles?"

Permit me to say that there is nothing in Dr. Smith's facts which should lead any man to doubt the existence of the germs of a communicable fever at the South which are frequently carried long distances to infect pastures and destroy the greater part of the native cattle that frequent them. There is no direct evidence in his reports ; he only attempts to show that Texas cattle are apparently and generally in good health, and that Northern cattle taken there die from the effects of change of climate, and from these premises, which I have shown are far from being satisfactorily established, he draws the conclusion that Texas cattle may be safely mingled with our Northern herds. He has never taken a drove of cattle in summer from Texas or the gulf regions of other States to the herding grounds in Kansas or Nebraska or Dakota, nor has he mixed such a drove with the native steers of Illinois or Iowa. A single experiment of this kind would probably remove the last remnant of his skepticism, and convince him of the danger of disregarding the practically unanimous testimony of thousands of experienced men.

It is not for me at this time to recount the hundreds of outbreaks of this disease now on record which have followed the thoughtless introduction of gulf coast cattle to the feeding grounds of the North. You cannot dispose of them with the remark that "there is no such rigorous analysis of facts as shows the conclusions drawn to be inevitable." They are inevitable, and for this reason : among the thousands and tens of thousands of cattle which have been affected at the North, and some of which have been located in every Northern State, not one case has occurred where it could not be shown that the animal had an opportunity to be infected by cattle from the distant South. Last year we had a report from away up in Dakota that cattle were dying with certain symptoms which reminded us of Texas fever, but not a word about Texas or Southern cattle. We wrote to know if animals had been introduced there from the South, and received a reply that thirty-one head had come from Mississippi, but as they were all well, it was not supposed they could have brought the disease. And so last year there were outbreaks in Kansas, Missouri, Illinois, Ohio, West Virginia, Virginia, New York and New Jersey, every one of which were traced to cattle from the infected districts of the South. Why is it that a disease with these characters never occurs at the North, unless Southern cattle have had access to the pastures?

This disease differs from all others sufficiently in its characters to make its diagnosis a matter of considerable certainty. I repeat it is only seen at the North in districts where Southern cattle have been introduced the same season; it affects Northern cattle taken to the South; the district from which cattle are dangerous corresponds exactly to that which is dangerous to Northern cattle. It matters not what we call the disease, the facts remain and this Association must accept them or lend its influence to the propagation of error. We are just now in need of laws to prevent the dissemination of this disease; to check the gradual advance of the district permanently infected with it; to exterminate it where possible; and we feel the need of the encouragement if not the active support of sanitarians generally, and especially of such influential sanitary associations as this. And it is only in the hope of increasing your interest in animal pathology, gentlemen, and of gaining your sympathy to aid in accomplishing a work that I can assure you is beset with many difficulties and discouragements, that I decided to appear before you and contest the conclusions of a fellow worker, who I doubt not is actuated by the same desire to establish the truth, that should be the guiding principle not only of every member of this Association, but of every scientific man.

In concluding, allow me to express the belief that it is too late to decide that Texas fever is a chimera; too late to try to explain away the terrible losses that have been traced only too certainly to the cattle from Southern ranges; too late to try to convince the Northern stock owner that sanitary regulations are not demanded for these animals; too late to try to wipe out the accumulated experience of a century. The hundreds who have lost their all through ignorance of the existence and characters of this plague, the thousands of square miles of territory that are ravaged almost annually, are enduring evidence of the reality of Texas fever.

### THE MEDICAL TREATMENT OF TEXAS CATTLE FEVER.

Among the most unsatisfactory of all diseases to treat are undoubtedly the contagious fevers, in which are included those fatal disorders which are so destructive to our domesticated animals. In this respect the medicine of man and the medicine of animals does not differ greatly. When a person has small-pox, diphtheria, scarlet fever or measles, the physician, if he be a wise one, no longer attempts to suddenly check the development or progress of these diseases. He knows that all such attempts are invariably failures, and that they usually result in actual harm to the patient. The disease is, therefore, allowed to run its course undisturbed so long as this course is a regular one, and the body of the patient is kept in the best possible condition to resist its ravages. Any tendency of the disease to unduly invade any of the vital organs is checked if possible; the circulation, the digestion, the excretions are kept in as good condition as may be; and last, though not least, the strength is preserved by stimulants and nourishing food. This is the most approved treatment of such fevers with man, and it is also the best treatment with animals; but unfortunately it requires the constant supervision of the trained physician or veterinarian as the case may be, and too often with animals this is impossible.

A radical cure of Texas fever, or any other contagious fever of animals, is at present beyond our ability, but we can do much to increase the chances of recovery from the greater part of these affections. To do this we must bear in mind the complications which are most injurious to the sick animal. One of the most important of these, in the disease under consideration, is the tendency to impaction or obstruction of the third stomach or maniplus by the drying of its contents. The presence of diarrhœa is not, as is generally supposed, any evidence that this impaction has not taken place, since the diarrhœa results from irritation in the intestines, and, therefore, back of the fourth stomach, while the impaction is in front of this organ when it is not affected. Another complication is the congestion of the gall-bladder and ducts connecting with it, which leads to tumefaction and constriction of the canal by which the bile reaches the intestine. As a result of this condition the whole apparatus is overloaded with this secretion of the liver, the pressure leads to absorption of the bile into the blood-vessels, and some of the worst characters of the disease have been attributed to this admixture of bile with the blood.

Both of these conditions are relieved by mild purgatives, and hence a physic which has for its basis salts, or oil, or calomel, has proved a rather successful method of treatment. In many parts of the country there are men who think that calomel is a specific, or that lard is a specific, because a few cases which they have treated with these purgatives have recovered. Still others consider green corn a specific, evidently because it too has a purgative effect; but it is neither so favorable nor certain in its action as salts, or as these combined with lard, oil or calomel. The following doses are suitable for administration to full grown cattle in this disease : Epsom salts, one pound, calomel, one drachm, given in about a quart of warm water ; or Epsom salts, one pound, linseed or castor oil, one pint, calomel, one drachm, mixed with warm water and molasses, and given as a drench.

Physic, however, is in no sense a specific. A few cases will recover without it, a larger number with it, many will die in spite of it. In all cases it should be repeated in a sufficient dose, and as often as may be necessary to keep the animal from becoming at all constipated.

Nourishing and easily digested food, such as ground oats or mixed bran and shorts, stirred with boiling water, must be given regularly, while corn and dry, hard food should be avoided.

Beyond this it may be doubted if treatment will pay in the hands of any but a veterinarian. I have thought that quinine in doses of 20 to 30 grains twice a day, for a full-grown animal, was beneficial. If such treatment is decided upon, chinoidine, which is much cheaper and answers the purpose very well with animals, may be substituted if the dose is doubled. When the bowels are very irritable, as is often the case, powdered chalk in half ounce doses, or subnitrate of bismuth half ounce, and chalk one-fourth ounce, will be found very beneficial. When the temperature is high, and the pulse rapid, I have sometimes been much pleased with tincture of aconite root in twenty drop doses three times a day.

In many parts of the South peach leaf tea is a favorite remedy, and as this contains some prussic acid, it may act as a sedative similar to aconite, but it is doubtful if in the doses usually administered it has this effect; but peach leaves have a laxative tendency which may be a better explanation of the favor in which they are held.

It must be remembered that with this, as with other contagious diseases, some outbreaks are much milder than others. That sometimes the majority of cases will recover without treatment, while at other times the majority will die with the very best of attention. Again, the class and condition of the affected animals have much to do with the result. Cows and Short-Horn steers usually die, common steers are less severely affected, while young cattle and calves generally recover.

The great point is to prevent this disease entirely, and as cattle only contract it by running on grounds which have been poisoned by other cattle from the permanently infected district of the South, this is usually not a difficult matter. This permanently infected district embraces Southeastern Virginia, North Carolina east of the mountains, South Carolina, Georgia, Florida, Alabama, Mississippi, Louisiana, the greater part of Texas, Indian Territory, and Southern Tennessee. It will be outlined with more detail in the reports of the United States Department of Agriculture, and to these I would refer all who contemplate the purchase of Southern cattle.

